# Enhanced Drug Delivery to Solid Tumors via Drug-Loaded Nanocarriers: An Image-Based Computational Framework

**DOI:** 10.3389/fonc.2021.655781

**Published:** 2021-06-24

**Authors:** Farshad Moradi Kashkooli, M. Soltani, Mohammad Masoud Momeni, Arman Rahmim

**Affiliations:** ^1^ Department of Mechanical Engineering, K. N. Toosi University of Technology, Tehran, Iran; ^2^ Department of Electrical and Computer Engineering, Faculty of Engineering, School of Optometry and Vision Science, Faculty of Science, University of Waterloo, Waterloo, ON, Canada; ^3^ Advanced Bioengineering Initiative Center, Multidisciplinary International Complex, K. N. Toosi University of Technology, Tehran, Iran; ^4^ Centre for Biotechnology and Bioengineering (CBB), University of Waterloo, Waterloo, ON, Canada; ^5^ Departments of Radiology and Physics, University of British Columbia, Vancouver, BC, Canada; ^6^ Department of Integrative Oncology, BC Cancer Research Institute, Vancouver, BC, Canada

**Keywords:** solid tumors, drug delivery, nanomedicine, drug-loaded nanocarriers, tumor penetration, image-based model, treatment efficacy

## Abstract

**Objective:**

Nano-sized drug delivery systems (NSDDSs) offer a promising therapeutic technology with sufficient biocompatibility, stability, and drug-loading rates towards efficient drug delivery to solid tumors. We aim to apply a multi-scale computational model for evaluating drug delivery to predict treatment efficacy.

**Methodology:**

Three strategies for drug delivery, namely conventional chemotherapy (one-stage), as well as chemotherapy through two- and three-stage NSDDSs, were simulated and compared. A geometric model of the tumor and the capillary network was obtained by processing a real image. Subsequently, equations related to intravascular and interstitial flows as well as drug transport in tissue were solved by considering real conditions as well as details such as drug binding to cells and cellular uptake. Finally, the role of periodic treatments was investigated considering tumor recurrence between treatments. The impact of different parameters, nanoparticle (NP) size, binding affinity of drug, and the kinetics of release rate, were additionally investigated to determine their therapeutic efficacy.

**Results:**

Using NPs considerably increases the fraction of killed cells (FKCs) inside the tumor compared to conventional chemotherapy. Tumoral FKCs for two-stage DDS with smaller NP size (20nm) is higher than that of larger NPs (100nm), in all investigate release rates. Slower and continuous release of the chemotherapeutic agents from NPs have better treatment outcomes in comparison with faster release rate. In three-stage DDS, for intermediate and higher binding affinities, it is desirable for the secondary particle to be released at a faster rate, and the drug with slower rate. In lower binding affinities, high release rates have better performance. Results also demonstrate that after 5 treatments with three-stage DDS, 99.6% of tumor cells (TCs) are killed, while two-stage DDS and conventional chemotherapy kill 95.6% and 88.5% of tumor cells in the same period, respectively.

**Conclusion:**

The presented framework has the potential to enable decision making for new drugs *via* computational modeling of treatment responses and has the potential to aid oncologists with personalized treatment plans towards more optimal treatment outcomes.

## Introduction

Encapsulation of drugs in nanoparticles (NPs) can enhance the delivery of therapeutic and diagnostic agents to tumors, and at the same time, reduce their accumulation in healthy tissue (1). In treatments, this issue results in improved local drug concentrations at disease sites while decreasing systemic toxicity. On the other hand, conventional small molecule compounds usually distribute randomly among all tissues (2). However, NPs using advantages such as enhanced permeability and retention effect (EPR) have provided moderate enhancements over small molecule therapeutics regarding patient survival and care (3). A qualitative analysis of the pharmacokinetics of NP-mediated doxorubicin (DOX) in patients with cancer was carried out by Gabizon et al. (4) comparing with an equal DOX dosage delivered in free form. The findings distinctly indicated that NP-mediated delivery is capable of decreasing plasma clearance and enhancing DOX concentration inside tumors (4). *In vivo* tests on human prostate carcinoma showed that NP-mediated DOX may improve treatment effectiveness because of decreased systemic elimination, enhanced penetration in tumor, and prolonged NP existence with a slow release rate of drug (5). The clinical advantages of NP-encapsulated DOX is also evaluated in patients with metastatic breast cancer (6). The results suggested that NP-mediated DOX was capable of significantly reducing cardiotoxicity and improving therapeutic efficacy. Nevertheless, despite multiple promising ideas and experimental outcomes, NPs do not commonly reach tumors efficiently in clinical trials (7). Successful drug delivery depends on the ability of NPs to deep penetration into tissue, achieve an efficient spatial distribution, ensure its proper binding affinity, and adequately release the drugs (8). However, progress in NPs will need a number of technical and physiological barriers to be realized and overcome, such as opsonization and nonspecific protein adsorption, non-specific uptake by cells and organs encompassing the immune system, targeting and penetrating the tumor microenvironment (TME), and gaining access to cancer cells for intracellular drug delivery (9). Physical circumstances influencing the function of NPs facing these obstacles are still understood imperfectly, and the nanomedicine lacks a detailed explanation of physical principles to guide logical NP designs that can overcome biological barriers in TME (1).

For most desirable efficiency, sufficient amounts of the therapeutic agents must reach tumors in order to eliminate cancer cells, and at the same time, they must not induce considerable side effects on normal tissues. Generally, relatively small NPs experience higher transvascular and interstitial transports (10). Investigation of the various functions of NPs with different physiological features in combating biological barriers has demonstrated that NPs require to be dynamically adapted to these obstacles, resulting in the emergence and development of multi-stage DDSs (11). In such a system, the NPs can change their size in response to stimuli at different stages (*e.g.*, initial size of 100 nm and secondary size of 10 nm) (12). Wong et al. (13) suggested a multi-stage system comprising the primary particle containing smaller secondary particles, which subsequently carry the therapeutic agent. Eventually, therapeutic agents contained in the secondary particles must be released to reach cancer cells. If an additional stage was included in the conventional NPs, it could improve drug distribution into the tumor and also tumor penetration, as well as increase treatment efficacy. The secondary particles release their cargo within the tumor, triggered by exposure to external (magnetic and electric fields, acoustic, *etc.*) or internal stimuli (TME properties such as enzymes, pH, *etc.*).

Since the introduction of the first model for chemotherapy, the use of mathematical and computational models has become widespread in examining different DDSs (14–19). These models can provide guidance on required composition and preparation methods for administration of DDSs. Various computational models have been employed for simulation of NSDDSs to examine efficacy, understand biological phenomena, and select optimal treatment plans. Based on the investigated spatial and temporal scales, these models are classified to discrete, continuous, and hybrid models (8). A summary is presented in **Table 1** of important studies conducted on employing mathematical modeling of drug-containing NPs for drug delivery to solid tumors (20–45).

**Table 1:** A summary of important studies conducted on employing mathematical modeling of drug-containing NPs for drug delivery to solid tumors.

Reference / Year	Subject	Geometry & simulation method	Findings	Study gap
El-Kareh and Secomb (20)/2000	Developing a mathematical model and applying it to compare the efficacy of different administration modes of both free DOX and thermo-sensitive liposome (TSL) encapsulated DOX.	A PK/PD model	Authors recommended that shorter injection duration might enhance treatment performance, and explored it by computational studies. The treatment outcome was predicated according to peak intracellular concentration over the whole treatment period. A comparison between bolus injection and continuous infusion demonstrated that duration of infusion had a great effect on the treatment outcome. Optimal duration is dependent on cellular pharmacokinetics. Drug release rate from non-TSLs is an effective parameter so that if this rate is optimized, the efficacy of non-TSLs is slightly fewer than continuous infusion.	-Spatial distribution is not considered.
Zhang et al. (21)/2009	Developing a mathematical model coupling heat and mass transfer to investigate spatiotemporal distributions of drug that are released from the liposome.	A 2D-0D* model & finite element method (FEM)	Compared to liposomes, diffusion of free drug plays a greater role in drug transport to tumor, as the free drug diffusivity is higher than that of liposomes. Hyperthermia alone only increases drug accumulation in the tumor periphery, and the TCs in the central area are barely damaged because of weak diffusion. Necrosis or apoptosis of the TCs can importantly affect the penetration of drug and must be taken into account in modeling of drug diffusion to precisely simulate the treatment effect. Combination of radio-frequency ablation and liposomal DOX delivery demonstrates more efficacious therapeutic result, particularly for larger tumors.	-Avascular model; -Normal tissue is not considered; -Real image of tumor is not considered.
Hendricks et al. (22)/2012	Presenting a multi-scale mathematical model of Liposomal DOX delivery for quantifying the role of parameters related to tumor and drug in drug delivery to solid tumors	A PK model	Authors illustrated that, for varying tumor transport features, there exist a regimen where liposomal and conventional DOX deliver identical amounts of dox to tumor cell nuclei. They also showed that liposome PKs and tumor deposition (which reflects vascular permeability) are highly variable.	-Spatial distribution is not considered.
Chauhan et al. (23)/2012	Investigating the effect of normalizing blood vessels of tumor for enhancing nanomedicine delivery in a size-dependent method	A 2D-1D model & FEM	Decreasing the vessel-wall pore size via normalization reduces the IFP in tumors, allowing small NPs to enter them more quickly. However, enhanced steric and hydrodynamic hindrances, also associated with smaller pores, make it more difficult for large NPs to enter tumors. It was suggested that smaller (∼12 nm) NPs are ideal for treating cancer because of their better penetration into the tumor.	-Real image of tumor is not considered; -Concentration equations are solved without taking into account the binding affinity and cellular internalization.
Gasselhuber et al. (24)/2012	Proposing a mathematical model for comparison of Conventional chemotherapy, TSLs, and stealth liposomes	A PK model	While stealth-DOX led to high concentrations in tumor in comparison with free-DOX, just a minor fraction was bioavailable, resulting in little cellular uptake. Optimum time constants of release for maximum cellular uptake for stealth-DOX and TSLs are obtained.	-Spatial distribution is not considered.
Zhan and Xu (25)/2013	Employing a mathematical modeling for TSL delivery of DOX to solid tumor	A 2D-0D model & FEM	The model was applied to idealized geometry of tumor, and comparisons have been performed between continuous infusion of DOX and TSL-mediated delivery. Authors illustrated that TSL-mediated delivery performs better in reducing concentration of drug in healthy tissues. Compared with direct infusion, TSL delivery results a much higher peak intracellular concentration of DOX, which may enhance fraction of killed cells in tumor thereby improving the treatment impact of the drug.	-Real image of tumor is not considered; -Avascular model; -Lack of capillary network and assumption of uniform vascular density in the tissue.
Stylianopoulos et al. (26)/2013	Developing a mathematical platform for NP delivery to solid tumors considering electrostatic interactions between the NPs and the negatively-charged vessel-wall pores.	A 2D-1D model & FEM	The model simulations offer that electrostatic repulsion has a small effect on the transcapillary transport of NPs. Conversely, electrostatic attraction generated even by small cationic charges can result in a two-fold enhancement in the transvascular flux of NPs into the tumor interstitium. For each size of NP, there exist an amount of charge density above which a sharp enhancement in transcapillary transport is simulated.	-Real image of tumor is not considered and instead, a mathematical model is used for angiogenesis; -Concentration equations are solved ignoring binding affinity and cellular internalization.
Kim et al. (27)/2013	Overviewing different mathematical frameworks of anti-cancer drug penetration into solid tumor	─ (Review paper)	Authors overviewed the state of mathematical modeling approaches that address phenomena regarding drug delivery. They described how different types of models were employed to predict spatial-temporal drug distributions in solid tumor, to simulate various approaches to overcome obstacles to drug delivery, or to optimize treatment programs. They also discussed how integration of *in silico* modeling with *in vivo* or clinical data can provide better tools to understand the drug transport process.	─
Stylianopoulos et al. (28)/2015	Employing mathematical modeling to examine the effect of drug features on the distribution and efficacy of NPs and also investigating two multi-stage NP delivery systems.	A 2D-1D tumor model & FEM	Adjusting the release kinetics and binding affinities of drug results in enhanced drug delivery. Smaller NPs have better treatment efficacy than bigger ones.	-Real image of tumor is not considered and instead, a 1D network is used for angiogenesis;
Stylianopoulos and Jain (29)/2015	Design considerations for nano-therapeutics in oncology.	─ (Review paper)	Authors evaluated different design parameters that can be regulated to optimize DDS, suggested specific design approaches that should optimize delivery to most tumors, and discussed under which circumstances active targeting would be advantageous.	─
Chou et al. (30)/2017	Developing a mathematical model of tumor according to interstitial fluid flow and particle transport to study the drug transport and cumulative concentrations in a tumor.	A 2D-0D tumor model & FEM	The efficacy of anti-cancer drug delivery was determined by the interplay of the microvascular density and NP size. All NPs and chemotherapeutic drugs have a limited concentration in the necrotic zone of tumor, where transport of drug is only through diffusion. Using NPs as anti-cancer drug carriers is generally a better option compared to molecular chemotherapeutic agent due to its higher therapeutic efficacy on tumor and lower damage to healthy tissue.	-Lack of capillary network; -Real image of tumor is not considered; -Concentration equations are solved ignoring binding affinity and cellular internalization.
Zhan and Wang (31)/2018	Investigating the convection-enhanced delivery of liposome containing DOX under different circumstances in an MRI-based brain tumor model.	A 3D-0D model & FVM	Liposomes are able to increase the accumulation and penetration of drug in the convection enhanced delivery treatment. Transport of liposome is affected by convection rather than diffusion. The effective delivery volume has nonlinear relation with the release-rate of drug.	-Avascular model; -Lack of capillary network and assumption of uniform vascular density in the tissue.
Shamsi et al. (32)/2018	Proposing a computational model for magnetically-assisted drug delivery approach to assess the penetration of drug into peritoneal tumors nodules and improve intraperitoneal (IP) chemotherapy.	A 2D-0D tumor model & FEM	A great enhancement in the intratumoral concentration of magnetic NPs compared to free drugs. The success of magnetic drug targeting in larger tumors (10–20 mm in size) is found to be significantly due to the strength of magnetic field and tumor-magnet distance while these two parameters are less important in small tumors.	-Avascular model; -Lack of capillary network and assumption of uniform vascular density in the tissue. -Concentration equations are solved ignoring binding affinity and cellular internalization.
Stylianopoulos et al (33)./2018	Reengineering the TME to enhance the efficacy of drug delivery from computational modeling to bench to bedside.	─ (Review paper)	Authors discussed the mechanics of both solid and fluid components of tumor, focusing on how they prevent the delivery of drug and create an abnormal TME that promotes tumor growth and resistance to treatment. They also provide strategies to re-engineer the TME by normalizing the vessels of tumor and the ECM to enhance the treatment of cancer. Eventually, they summarized different mathematical approaches that have provided insights into the physical obstacles against efficient cancer treatment and suggested novel methods to overcome these impediments.	─
Huang et al. (34)/2019	Presenting a mathematical modeling for spatial–temporal distribution of chemotherapy drug in TSL-mediated DDSs	A PK/PD model	Authors demonstrated that complicated relationships between the related factors (various chemotherapy drugs, release rate constants, and heating duration) and the predicted treatment result, making it difficult to identify the best parameter set. a model-based optimization approach is presented to overcome this challenge. Optimization showed that the best result would be obtained with a low drug release rate at physiological temperature, combined with a moderate to high release rate at mild hyperthermia and 1 h heating post- injection.	-Spatial distribution is not considered;
Rezaeian et al. (35)/2019	IP injection of TSL DOX with the triggered release by mild hyperthermia caused by high intensity focused ultrasound.	A 2D-0D tumor model & FEM	Using TSL-DOX delivery is efficacious than conventional chemotherapy. Adjusting the TSL size must be carried out according to the vessel wall permeability. Smaller TSLs have better treatment efficacy. TSL-DOX delivery system in smaller tumors is less beneficial compared to larger ones.	-Avascular model; -Lack of capillary network and assumption of uniform vascular density in the tissue.
Shamsi et al. (36)/2019	A review of computational modeling of nano-engineered DDSs.	─ (Review paper)	Authors investigated different theoretical modeling approaches as influential tools to furnish future design and development of DDSs.	─
He et al. (37)/2019	Developing a mathematical modeling to analyze nanomedicine distributions in solid tumors	A PK model	Authors quantified the effect of influencing parameters on the efficacy of tumor delivery, the magnitude of heterogeneous distribution, and the EPR effect. They also compared the spatial distributions of the NPs and the free drugs within tumors. The model predicted high degrees of distributional heterogeneity for both NPs and free drugs. They found that diffusion coefficient of NPs was the most efficient factor in decreasing the NPs distributional heterogeneity but it has moderate impact on the free drugs.	-Real image of tumor is not considered; -Spatial distribution is not considered; -Lack of capillary network and assumption of uniform vascular density in the tissue.
Dogra et al. (38)/2019	Overviewing different mathematical modeling about application of nanomedicine in cancer treatment.	─ (Review paper)	Authors provided an overview on mathematical modeling works that have been applied towards a better insights of nano-bio interactions for enhancing the efficacy of drug delivery to tumor.	─
Tehrani et al. (39)/2020	Conducting numerical simulation to investigate the impacts of diffusion of MNPs on microwave ablation treatment.	A 2D-0D tumor model & FEM	Injection process has an essential impact on distribution of MNPs. Sufficient diffusion time can enhance the ablation zone after thermal therapy. Balance between diffusion time and size of MNPs can enhance the efficacy of therapy.	-Real image of tumor is not considered; -Avascular model; -Drug transport equations are solved ignoring binding affinity and cellular internalization.
Wirthl et al. (40)/2020	Presenting a multi-phase tumor growth model to examine NP delivery to solid tumors	A 2D-0D tumor model & FEM	This study allows investigation of the properties and of the limitations of NP delivery to solid tumors, which currently complicate the translation of NP therapy to a clinical trials	-Real image of tumor is not considered; -Drug transport equations are not investigated.
Wijeratne and Vavourakis (41)/2020	Proposing a mathematical framework of dynamic growth of solid tumor, drug delivery, and angiogenesis.	A 3D-1D tumor model & FEM	This model allows for drug features (e.g., size and binding affinity) to be explicitly defined, thus facilitating investigation into the interaction between the changing TME and cytotoxic and NP drugs. They predict a heterogeneous distribution of NPs after delivery; that NPs need a ECM with high porosity to cause tumor regression; and that transcapillary fluid velocity is dependent on porosity of ECM, and implicitly on the drug size.	-Real image of tumor is not considered.
Dogra et al. (42)/2020	Conducting sensitivity analysis to characterize the effective parameters on low delivery of NP to tumor and high off-target accumulation of NPs by whole-body NP pharmacokinetics	Physiologically based PK model	Degradation rate of NPs, size of NPs, blood viscosity of tumor, vascular fraction of tumor, and tumor vascular porosity of tumor are effective factors in governing kinetics of NPs within the interstitial space of tumor.	-Real image of tumor is not considered; -Simulation is performed without taking into account the binding affinity and cellular uptake.
Shojaee et al. (43)/2020	Effect of NP size, magnetic intensity, and tumor distance on the distribution of the MNPs in a TME	A 2D-2D tumor model & FEM	Magnetic field and size changes has a moderate impact on the drug penetration to the tumor. The dense ECM, elevated IFP, and the availability of capillary network have negative influences on the MNP distribution. The size and the magnetic field are the two most promised factors for enhancing the convection term in the tumor area.	-Real image of tumor is not considered; -Simulation is performed without considering the binding affinity and cellular uptake.
Stillman et al. (44)/2020	Presenting *in silico* modeling of nanomedicine for cancer, across scales and transport obstacles.	─ (Review paper)	Authors investigated latest outcomes in multi-scale modeling of NP transport obstacles, as well as existing software packages, with the goal of focusing the wider research community in building a common computational platform that able to overcome some of the current barriers facing effective design of NPs.	─
Moradi Kashkooli et al. (45)/2021	A review of different mathematical modeling approaches for NSDDSs.	─ (Review paper)	Investigation of various issues regarding the use of NPs as vehicles of anticancer drug delivery: specifically, administration into the circulation system, transvascular transport, distribution in the extracellular matrix, cellular internalization, and release of drug from NPs.	─

*A 2D-0D means that the geometry of tumor and microvascular network are considered 2-dimensional and 0-dimentional (avascular), respectively.

As seen in **Table 1**, there exist a number of gaps in the literature regarding usage of drug-containing NPs for delivery of drug to solid tumors. With this motivation, in the present study, the main contribution is to apply a computational model of three DDSs, namely (*i*) a conventional chemotherapy system (one-stage DDS) and (*ii*) a conventional two-stage DDS containing NP and chemotherapy (two-stage DDS), and (*iii*) a three-stage DDS containing a primary NP, a secondary NP, and the chemotherapy; on a real image of vascularized tumor. To this end, intravascular and interstitial fluid flows as well as drug transport equations are solved by considering actual conditions in tumor. Then, various parameters, NP size, binding affinity of drug ligands to the receptors of cells, and the kinetics of release rate, are studied to determine their effects on treatment efficacy. NPs employed in this study could for instance be drug-loaded nanocarriers, liposomes, or magnetic NPs. Additionally, the impact of successive treatment cycles is numerically examined considering tumor recurrence between two consecutive treatments for three investigated DDSs.

## Material and Methods

In this section, a detailed description of mathematical models and their assessment are investigated. First, the mathematical equations are presented, including equations governing the interstitial fluid flow, solute transport and cellular uptake of chemotherapeutic agents, and NP delivery system. Then, parameters of model, relevant physical and transport properties, and NP-related calculations are also provided. Finally, numerical methods to assess the mathematical models, model parameters, boundary conditions (BCs), input images as geometry, computational domain, as well as solution strategy are presented.

### Delivery Mechanisms

Two well-known delivery mechanisms, namely chemotherapeutic delivery and delivery through drug loaded NP, are considered in the present study. In the following, their mechanisms are described in detail.

After intravenous (IV) injection, the chemotherapeutic drugs (here, DOX) are transported to the tumor site through the blood vessels. Subsequently, they pass *via* the tumor vessel wall and travel the remaining distance from the vessel wall to the cancer cells. In tumor ECM, free molecules of drug agents can bind to receptors of the cells, unbind or get internalized (28, 46).

**Figure 1:**
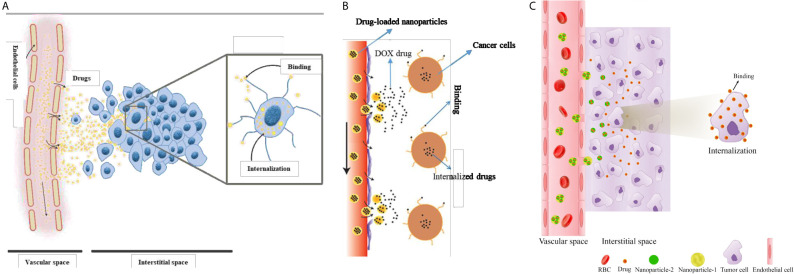
Schematic of drug delivery mechanisms considered in the current study. **(A)** one-stage DDS or conventional chemotherapeutic delivery, **(B)** two-stage DDS (i.e., NP delivery), and **(C)** three-stage DDS.

In general, only a small fraction of an injected drug reaches tumor tissue, the remaining being cleared from the body. One possible way to overcome this problem is to target drug delivery by encapsulating the anticancer drug in a nanocarrier (47). In such a system, drugs which encapsulated inside NPs are injected into the blood, and subsequently, NP characteristics cause them to be released in accordance with a pre-designed controllable procedure. NP formulations have benefits over commonly used chemotherapies since they can combine many diagnostic and therapeutic factors (48). They are also associated with significantly lower side effects, owing to their capacity for optional accumulation in tumorous tissue. To eradicate TCs, NPs have to first reach the tumor *via* the vascular system, then extravasate from the relatively leaky regions of the microvessels into the TME. Subsequently, NPs release their cargo in a controlled manner, while staying put at the ECM. Drug molecules diffuse into the tumor slightly more than into normal tissue and can bind to cancer cells and/or TME components eventually, becoming internalized by cells (28). A schematic of drug delivery mechanisms considered in the current study is shown in **Figure 1**.

### Governing Equations

The mathematical models for delivery of drugs to solid tumors consist of equations as:

- Mass and momentum conservation for interstitial fluid flow;
- Mass transport for the free, bound, and internalized drug;
- Mass transport for nano-encapsulated drug; and
- Treatment efficacy for intracellular drug concentration.

Detailed descriptions of each aspect of mathematical modeling are provided next.

The dynamics process in drug delivery includes binding and unbinding of drug agent ligands with receptors of the cells at the rates of K_ON_ and K_OFF_, respectively; exchange of drug between capillary network and interstitium, and influx/efflux of drugs from interstitium to TCs, internalization of drugs to cellular space, and finally cell-killing by drug agents. The cell-killing rate is governed by a model according to the predicted intracellular concentration of anticancer drugs. In the case of two-stage and three-stage DDSs, additional equations describing the transport of NPs in ECM are required. Mathematical modeling of fluid flow and solute transport in interstitium, convection-diffusion-reaction (CDR) modeling of drug transport in the extracellular space (for conventional chemotherapy, two-stage, and three-stage DDSs), as well as FKCs and tumor-cell survival equations are presented in the following sections, respectively.

#### Fluid Flow in Interstitium

First, the interstitial fluid flow equations are solved to provide the basic biomechanical environment for transport of drug. Darcy equation, which demonstrates the relationship between interstitial fluid velocity (IFV) and interstitial fluid pressure (IFP), is employed to describe interstitial fluid flow in a porous environment as follows (16):

(1) $$v_{i}=-\kappa \nabla P_{i}$$

where *v_i_* and κ are the IFV and the hydraulic conductivity. Considering the presence of source/sink terms in biological tissues, the continuity equation is modified as (7):

(2) $$\nabla \cdot v_{i}=\phi_{B}-\phi_{L}$$

in which *ϕ_B_* is the rate of fluid flow from the microvessels to the extracellular matrix (ECM) and and *ϕ_L_* is the rate of fluid flow from ECM to lymph system, defined as (7):

(3) $$\phi_{B}=L_{p}\left(\frac{S}{V}\right)\left(P_{B}-P_{i}-\sigma_{s}\left(\pi_{B}-\pi_{i}\right)\right)$$

(4) $$\phi_{L}=L_{PL}{\left(\frac{S}{V}\right)}_{L}\left(P_{i}-P_{L}\right)$$

in which *S/V* demonstrates the surface area per unit volume of microvessels. $L_{PL}{\left(\frac{S}{V}\right)}_{L}$ and *P_L_* are the lymphatic filtration coefficient and lymphatic pressure, respectively.

Combining Eq. (2) with Eq. (1), we arrive at:

(5) $$-\kappa \nabla^{2}P_{i}=\phi_{B}-\phi_{L}=L_{p}\left(\frac{S}{V}\right)\left(P_{B}-P_{i}-\sigma_{s}\left(\pi_{B}-\pi_{i}\right)\right)-L_{PL}{\left(\frac{S}{V}\right)}_{L}\left(P_{i}-P_{L}\right)$$

#### Transport of Drug in the Interstitium

The comprehensive model for drug transport in the interstitial space includes:

- Transport in ECM by diffusion and convection mechanisms,
- Transport across microvessels by diffusion and convection mechanisms, and
- Binding to cells and internalization.

The drug transport equation for biological tissue can be written as (18):

(6) $$\frac{\partial C}{\partial t}=\nabla \cdot [D_{eff}\nabla C]-\nabla \cdot [v_{i}C]+(\Phi_{B}-\Phi_{L})$$

in which Ф_B_ is the drug transport rate through the microvessels into the interstitial space, and Ф_L_ is the drug transport rate from interstitial space into the lymph system. Ф_B_ is defined according to Patlak’s model, as (18, 49):

(7) $$\Phi_{B}=\phi_{B}(1-\sigma_{f})C_{p}+\frac{PS}{V}(C_{p}-C_{f})\frac{P_{e}}{e^{Pe}-1}$$

(8) $$Pe=\frac{\phi_{B}(1-\sigma_{f})}{P\frac{S}{V}}$$

*Pe* demonstrates the Peclet number, *σ_f_* is the coefficient of filtration reflection, *P* represents the permeability coefficient of microvessels, and *C_p_* is the injected drug concentration.

The drug transport rate through lymphatic microvessels has been considered only in healthy tissue as (18, 49):

(9) $$\Phi_{L}=\phi_{L}C$$

A bolus injection of chemotherapeutic agents, representing the initial vascular concentration of the drug, is modeled as (50):

(10) $$C_{p}=C_{0}\exp(-t/k_{d})$$

where *C_0_* and *k_d_* are the initial concentration and blood circulation decay, respectively.

#### Convection-Diffusion-Reaction Modeling of Drug Transport in the Interstitium

**Figure 2:**
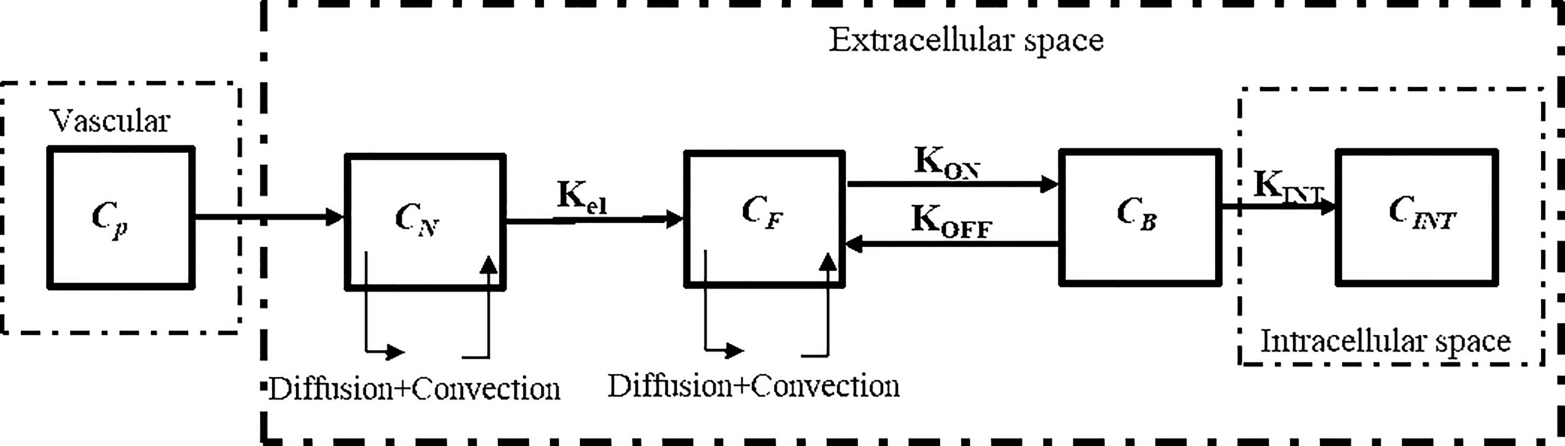
Block diagram of the current study for computational modeling of drug transport of two-stage DDS.

The drugs exist in different forms as: NPs in the interstitial space (*C_N_*), free drugs in the interstitial space (*C_F_*), bound drugs (*C_B_*), and intracellular drugs (*C_INT_*) (20, 49). In tissues, with regards to the existence of mass flow source/sink, a system of equations, which is called CDR equations, is utilized to represent the process of drug delivery. The general block diagram of the model of current study considering NPs and chemotherapeutic drugs for two-stage DDS is shown in **Figure 2**.

##### Conventional Chemotherapy

The CDR equations for conventional chemotherapy are as follows (17, 41):

(11) $$\begin{array}{ll} \frac{\partial C_{F}}{\partial t}=-v\nabla C_{F}+D_{eff}\nabla^{2}C_{F}-\frac{1}{\varphi }K_{ON}C_{rec}C_{F}+K_{OFF}C_{B}+(\Phi_{B}-\Phi_{L}) & Free\;drug\\ \frac{\partial C_{B}}{\partial t}=\frac{1}{\varphi }K_{ON}C_{rec}C_{F}-K_{OFF}C_{B}-K_{INT}C_{B} & Bound\;drug\\ \frac{\partial C_{INT}}{\partial t}=K_{INT}C_{B} & Drug\text{ internalized into }the\;cell \end{array}$$

in which *C_F_* demonstrates free drug concentration, *C_B_* bound drug concentration, *C_INT_* internalized drug concentration, and *C_rec_* is the concentration of cell-surface receptor. In this equations, *v* is the IFV, D is the diffusion coefficient, K_ON_ is the association rate, K_OFF_ is disassociation rate, K_INT_, represents the cellular internalization; and *φ* demonstrates the tumor volume fraction available for the drugs.

##### Two-Stage Drug Delivery System

Systemically administered NPs, as demonstrated in **Figures 1** and **2**, are transported to tumor sites through the circulation system, undergo transvascular extravasation followed by distribution in the interstitial space, and are finally delivered to cancer cells. For transport of an NP containing chemotherapeutic agents (*C_N_*), the system of equations is adjusted thus (51):

(12) $$\begin{array}{ll} \frac{\partial C_{N}}{\partial t}=-v_{i}\nabla C_{N}+D_{N}\nabla^{2}C_{N}-K_{rel}C_{N}+(\Phi_{V}-\Phi_{L}) & Nano-carrier\\ \frac{\partial C_{F}}{\partial t}=\alpha K_{rel}C_{N}-v_{i}\nabla C_{F}+D\nabla^{2}C_{F}-\frac{1}{\varphi }K_{ON}C_{rec}C_{F}+K_{OFF}C_{B} & Free\;drug\\ \frac{\partial C_{B}}{\partial t}=\frac{1}{\varphi }K_{ON}C_{rec}C_{F}-K_{OFF}C_{B}-K_{INT}C_{B} & Bound\;drug\\ \frac{\partial C_{INT}}{\partial t}=K_{INT}C_{B} & Internalized\;drug \end{array}$$

in which *K_rel_*, *D_N_*, and *α* are respectively the drug release rate from the carrier, its diffusion coefficient, and the number of chemotherapy molecules contained in the nanocarrier.

##### Three-Stage Drug Delivery System

Three-stage NP drug delivery system is an efficient option to overcome the barriers of two-stage DDS in ECM. The system of equations for three-stage DDS, as demonstrated in **Figure 1C**, is as following (32):

(13) $$\begin{array}{ll} \frac{\partial C_{N1}}{\partial t}=-v_{i}\nabla C_{N1}+D_{N1}\nabla^{2}C_{N1}-K_{rel1}C_{N1}+(\Phi_{V}-\Phi_{L}) & Nano-carrier\;1\\ \frac{\partial C_{N2}}{\partial t}=\alpha K_{rel1}C_{N1}-v_{i}\nabla C_{N2}+D_{N2}\nabla^{2}C_{N2}-K_{rel2}C_{N2} & Nano-carrier\;2\\ \frac{\partial C_{F}}{\partial t}=\beta K_{rel2}C_{N2}-v_{i}\nabla C_{F}+D\nabla^{2}C_{F}-\frac{1}{\varphi }K_{ON}C_{rec}C_{F}+K_{OFF}C_{B} & Free\;drug\\ \frac{\partial C_{B}}{\partial t}=\frac{1}{\varphi }K_{ON}C_{rec}C_{F}-K_{OFF}C_{B}-K_{INT}C_{B} & Bound\;drug\\ \frac{\partial C_{INT}}{\partial t}=K_{INT}C_{B} & Internalized\;drug \end{array}$$

in which *C_N1_* and *C_N2_* are the primary and secondary NP concentrations, respectively. *K_rel1_* is the rate constant for the release of the secondary NP from the primary one and *K_el2_* the rate constant for the drug release from the secondary NP. *α* and *β* are the number of secondary NPs released by the primary and drug particles released by the secondary NP, respectively.

#### Fraction of Killed Cells

Despite its cardiotoxicity, the drug DOX is frequently employed in tumor treatment. DOX is a standard-of-care, DNA-damaging agent employed in the treatment of multiple tumors (*e.g.*, bladder, breast, and lung cancers). Using the internalized drug concentration, the efficacy of drugs was calculated according to the empirical equation obtained for DOX (52). The FKCs parameter is defined, as (53):

(14) $$FKC=1-S_{F}=1-\exp(-\omega \cdot C_{INT})$$

in which *S_F_* is the fraction of cells remaining after treatment and *ω* is a fitting parameter specified for DOX based on the results of experiments (54).

#### Tumor-Cell Survival

The number of TCs after a period of time (*n_i_*), which is obtained through Gompertz’s model, depends on intervals between chemotherapy sessions (*t*) (55). Gompertz equation is a function of three parameters (as demonstrated in Eq.)15(): the number of TCs surviving after the *i* ^th^ treatment (*N_i_*), the number of saturated cells after a very long period (*N_∞_*), and eventually the rate of tumor progression (*b*).

(15) $$n_{i}(t)=N_{i}\;exp\left\{Ln\;\left(\frac{N_{\infty }}{N_{i}}\right)\left[1-exp\left(-bt\right)\right]\right\}$$

The number of surviving TCs after each therapeutic phase will be examined as a criterion of treatment efficacy evaluation. This criterion is obtained by using the FKCs according to Eq. (14). The number of remaining TCs as a result of the difference between the number of cells after (*N_i_*) and before (*N_0_*) treatment will also be examined as a criterion for evaluating treatment efficacy. In the present model, *S_F_* is also defined, as (30):

(16) $$S_{F}=\frac{N_{i}}{N_{0}}$$

The initial numbers of TCs here, adopted from York et al. (55), are *N_0_* = 5 × 10^9^, *N_∞_* = 3.1 × 10^12^, and *b* = 0.0283 month^-1^. The number of cells for healthy tissue was considered to be *N_1_* = 4.64 × 10^12^ (55). The cell number is assumed to depend on tumor volume; *i.e.*, when the cell number decreases, the tumor shrinks (*R* reduces). The ratio of the density of healthy tissue to the density of TC is considered to be 0.2 (56), and it is also assumed that the microvascular density distribution in the computational field does not change after each treatment. In addition, the regrowth of healthy tissue cells was examined by using Eq. (15) with the assumption that the growth rate (*b*) of healthy tissue is half that of the tumor (30).

### Model Parameters

#### Interstitial and Drug Transport Parameters

**Tables 2** and **3** demonstrate both the interstitial and DOX drug transport parameters, respectively, including both tumor and healthy tissues. **Table 4** represents the baseline state parameters for NSDDS for 20 nm particle size and 200 nm vessel-wall pore (VWP) size.

**Table 2:** Parameters of interstitial transport used in numerical simulations.

Parameter	Unit	Description	Value	Ref.
*π_B_*	[mmHg]	Oncotic pressure of microvessels	20 (Normal)	(57)
			20 (Tumor)	
*π_i_*	[mmHg]	Oncotic pressure of interstitial fluid	10 (Normal)	(57)
			15 (Tumor)	
*σ_s_*	–	Coefficient of average osmotic reflection	0.91 (Normal)	(57)
			0.82 (Tumor)	
*L_p_*	[cm/((mmHg)*s)]	Hydraulic conductivity of the microvessel wall	0.36×10^-7^ (Normal)	(57)
			2.8×10^-7^ (Tumor)	
*L*_pL_ *S*_L_ */V*	[1/(mmHg*s)]	Coefficient of Lymph filtration	1.33×10^-5^ (Normal)	(58)
			0 (Tumor)	
κ	[cm^2^/(mmHg*s)]	Hydraulic conductivity of interstitium	8.53×10^-9^ (Normal)	(58)
			4.13×10^-8^ (Tumor)	
P_L_	[Pa]	Hydrostatic pressure of lymph vessels	0	(58)

**Table 3:** Parameters for chemotherapy drug (DOX) applied to computational modeling.

Parameter	Unit	Description	Value	Ref.
*D*	[m^2^/s]	Coefficient of diffusion	1.58×10^-10^ (Normal)	(58)
			3.40×10^-10^ (Tumor)	
*P*	[m/s]	Microvessel permeability coefficient	3.75×10^-7^ (Normal)	(58)
			3.00×10^-6^ (Tumor)	
*σ_f_*	–	Filtration reflection coefficient	0.35	(52)
K_ON_	[m^3^/(mole s)]	Binding rate constant	15	(32)
K_OFF_	[1/s]	Unbinding rate constant	8×10^-3^	(32)
K_INT_	[1/s]	Internalization rate constant	5×10^-5^	(32)
*φ*	–	Volume fraction of tumor available to drugs	0.4	(32)
C_rec_	[M]	Cell-surface receptors concentration	1×10^-5^	(32)
K_d_	[Min]	Half-life of drug in plasma	6	(32)
*ω*	[m^3^/mole]	Survival constant of cancer cells	0.6603	(51)

**Table 4:** Parameters of baseline state for NP drug delivery for 20 nm particles and 200 nm VWP size.

Parameters	Unit	Description	Value	Ref.
D	[m^2^/s]	Diffusion	7×10^-12^	(30)
*φ*	*-*	Volume fraction of tumor available to drugs	0.05	(32)
K_ON_	[m^3^/(mole s)]	Binding rate constant	15	(32)
K_OFF_	[s^-1^]	Unbinding rate constant	8×10^-3^	(32)
K_INT_	[s^-1^]	Cellular uptake rate constant	5×10^-5^	(32)
K_el_	[s^-1^]	Release rate constant	2.1×10^-6^	(32)
K_d_	[min]	Blood circulation decay constant	1320	(32)
*α*	–	Number of particles in the NP carrier	20	(32)
C_rec_	[M]	Concentration of cell-surface receptors	1×10^-5^	(32)

#### NP-Related Calculations

The hydraulic conductivity, vascular permeability, and reflection coefficient are calculated for the NPs by using the theory of particle transport through cylindrical pores (28, 59):

(17) $$L_{p}=\frac{\gamma r_{o}^{2}}{8\mu L}$$

(18) $$P=\frac{\gamma HD_{o}}{L}$$

(19) $$\sigma_{f}=1-W$$

in which *γ* is the fraction of the surface area of a porous vessel-wall, *r_o_* is the pore radius, *η* is the viscosity of water at 310 K, and *L* is the vessel-wall thickness. *D_o_* represents a particle diffusion coefficient in a free solution at 310 K, given by the Stokes-Einstein relationship as in (53, 59):

(20) $$D_{o}=\frac{K_{b}T}{6\pi \eta r_{s}}$$

in which *K_b_*, *T*, and *r_s_* are the Boltzmann constant, the temperature, and the diffusing particle radius, respectively.

*H* and *W* are diffusive and convective hindrance factors, respectively, and related to hydrodynamic and electrostatic interactions. Neglecting electrostatic interactions (*E*=0), *H* and *W* are reduced to (53, 59):

(21) $$H=\frac{6\pi F}{K_{t}}$$

(22) $$W=\frac{F(2-F)K_{s}}{2K_{t}}$$

where *F* is the partition coefficient and is defined as (53, 60):

(23) $$F={\left(1-\lambda \right)}^{2}$$

in which *λ* is the ratio of drug particle size to the VWP size, as follows (53, 59):

(24) $$\lambda =\frac{r_{s}}{r_{o}}$$

*K_t_* and *K_s_* coefficients in Eqs. (21) and (22) are given by (53, 59):

(25) $$\left(\begin{array}{l} K_{t}\\ K_{s} \end{array}\right)=\frac{9}{4}\pi^{2}\sqrt{2}{\left(1-\lambda \right)}^{-\frac{5}{2}}\left[1+\sum_{n=1}^{2}\left(\begin{array}{l} a_{n}\\ b_{n} \end{array}\right){\left(1-\lambda \right)}^{n}\right]+\sum_{n=0}^{4}\left(\begin{array}{l} a_{n+3}\\ b_{n+3} \end{array}\right)\;\lambda^{2}$$

Parameter values used in the Eqs. (17) to (25) are demonstrated in **Table 5**.

**Table 5:** Parameter values used for NP-related calculations.

Parameter	Description	Value	Ref.
*L*	Vessel-wall thickness	5×10^−6^ m	(31)
*η*	Water viscosity at 310K	7×10^−4^ Pa∙s	(31)
*γ*	Fraction of surface area of vessel-wall occupied by pores	1×10^−4^ [-]	(51)
*a*_1_	1^st^ coefficient for *K_t_*	-73/60 [-]	(59)
*a*_2_	2^nd^ coefficient for *K_t_*	77.293/50.400 [-]	(59)
*a*_3_	3^rd^ coefficient for *K_t_*	-22.5083 [-]	(59)
*a*_4_	4^th^ coefficient for *K_t_*	-5.617 [-]	(59)
*a*_5_	5^th^ coefficient for *K_t_*	-0.3363 [-]	(59)
*a*_6_	6^th^ coefficient for *K_t_*	-1.216 [-]	(59)
*a*_7_	7^th^ coefficient for *K_t_*	1.647 [-]	(59)
*b*_1_	1^st^ coefficient for *K_s_*	7/60 [-]	(59)
*b*_2_	2^nd^ coefficient for *K_s_*	-2.227/50.400 [-]	(59)
*b*_3_	3^rd^ coefficient for *K_s_*	4.0180 [-]	(59)
*b*_4_	4^th^ coefficient for *K_s_*	-3.9788 [-]	(59)
*b*_5_	5^th^ coefficient for *K_s_*	-1.9215 [-]	(59)
*b*_6_	6^th^ coefficient for *K_s_*	4.392 [-]	(59)
*b*_7_	7^th^ coefficient for *K_s_*	5.006 [-]	(59)

### Model Geometry and Boundary Conditions

**Figure 3:**
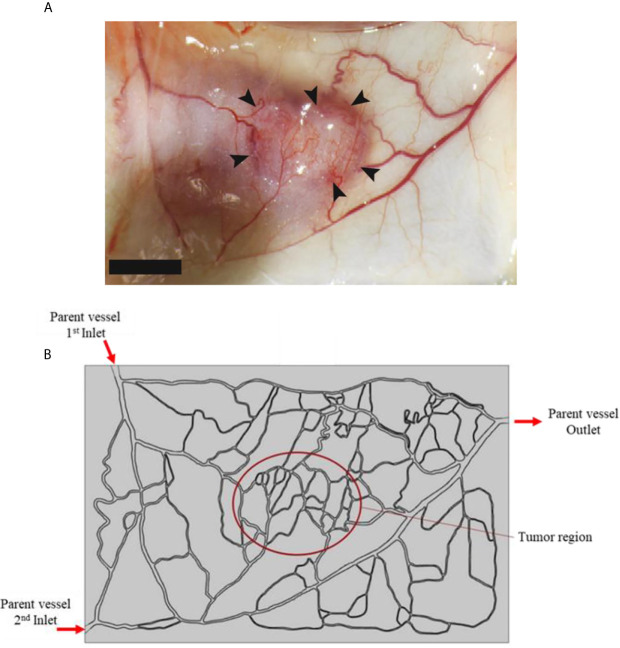
**(A)** Real image of tumor, and **(B)** computational field considered in numerical simulation which is obtained by image-processing of realistic image.

In this study, a tumor model (as shown in **Figure 3A**) is employed as an input geometry based on real image of a tumor with a capillary network surrounded by healthy tissue, extracted from Roudnicky et al. (60). About the circumstance of this input image, it should be mentioned that this type of tumor is inoculated in a mice by injecting human A431 squamous cell carcinoma (SCC) cells (60). This image was taken about 12 days after tumor inoculation. In fact, 2 days after inoculation, thrombospondin-2 (TSP2), an anti-angiogenic matricellular protein that inhibits tumor growth and angiogenesis, was injected for 10 days and subsequently this image was taken. After image-processing, a computational field is considered with the existence of a tumor in the middle of the domain as well as the parent vessels (**Figure 3B**).

Two boundaries are assumed in the computational field: the inner boundary (between the tumor and the normal tissue), and the outer boundary (at the outer edges of computational field). For the inner boundary, the continuity BC is considered for IFV, IFP, as well as concentration and its flux. For the outer boundary, in which the IFP is constant, the Dirichlet BC is used for fluid flow, and the open boundary is employed for concentration (50). The input and output pressure amounts for the parent vessels are selected as: *P*_Inlet,1 =_ 25 mmHg, *P*_Inlet,2 =_ 25 mmHg, and *P*_Outlet_ =10 mmHg, according to realistic physiological conditions reported in literature (50, 61).

### Solution Strategy

There exist two distinct phases for solving the present problem: steady-state and transient. Calculations related to blood flow in a model with a discretized capillary network in the computational field provides a system of non-linear equations. Thus, an iterative approach was applied to solve the fluid flow equations. The blood flow and interstitial fluid flow were solved concurrently, where IBP and IFP were coupled *via* Starling’s equation (Eq. (3) in supporting file). Obtained values for IBP, IFP, and IFV were employed for solving the transient equations of CDR to achieve different drug concentrations (*C_N1_*, *C_N2_*, *C_F_*, *C_B_*, and *C_INT_*) as well as FKCs.

After image-processing, geometries were meshed and analyzed utilizing the COMSOL Multiphysics software-version 5.5a. The coupled nonlinear set of the above-mentioned governing equations and also the BCs were assessed through FEM. A segregated approach is applied to solve the equations with the time-step of 0.1 [s] and relative tolerance of 0.001. A six-fold drop of residuals is chosen as the criterion for convergence. For solving drug delivery equations in vascularized tumors, a Core (TM) i24 CPU @ 3 GHz with 32 GB RAM system is used.

## Results and Discussion

Characteristics of tumors are determinant factors in transport of drug and final efficacy of treatment. Since chemotherapy drugs are carried by the circulatory system, the tumor vasculature features play an important act in delivery of drug. In the present study, a tumor model incorporating details of capillary network distribution is employed to evaluate the impact of heterogeneous distribution of capillary network on delivery of drug. This is followed by studies of transport of drug in tumors with microvascular density (MVD). First, the results of chemotherapeutic agent delivery for a case study, extracted from real image of tumor, are proposed, and different parameters are investigated in detail. Subsequently, results of computational modeling for delivery of NPs are presented.

### Validation of the Results

**Figure 4:**
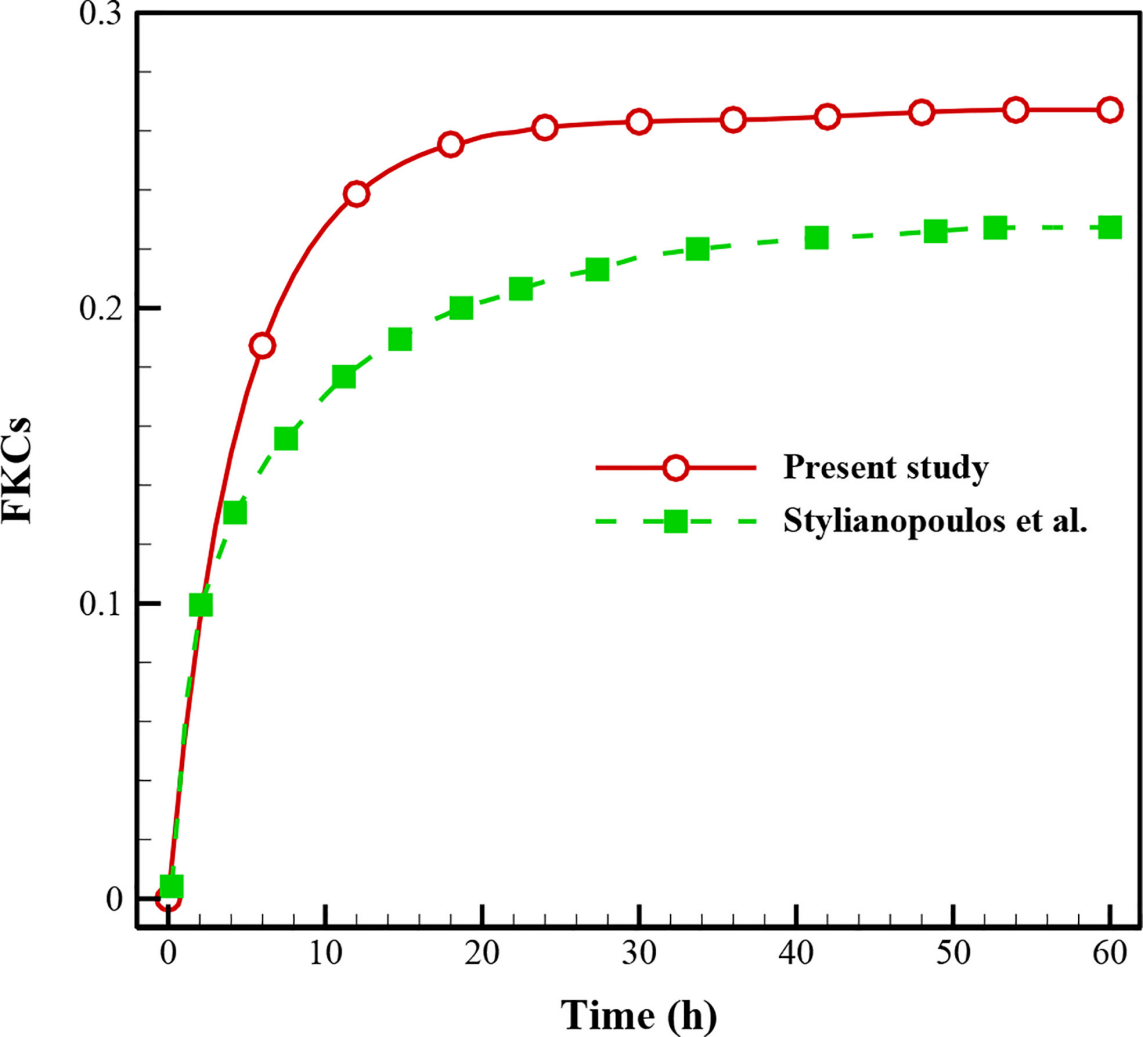
Comparison of the results with previously published study (28) using FKCs over time.

In this study, validation is performed with the same governing equations as the literature (28) for the FKCs over time (**Figure 4**). As is clear, there is a correspondence between the results of the present study and those in the literature so that by considering the real geometry and physics, the FKCs has a similar trend. However, considering similar conditions, its value has about 4% difference, due to differences in tumor geometry, computational domain, and structure of capillary network. One should note the 2D geometry of the tumor and capillary network investigated in this study, while Stylianopoulos et al. (28) utilized 1D for the capillary network.

### Baseline Model Analysis

**Figure 5:**
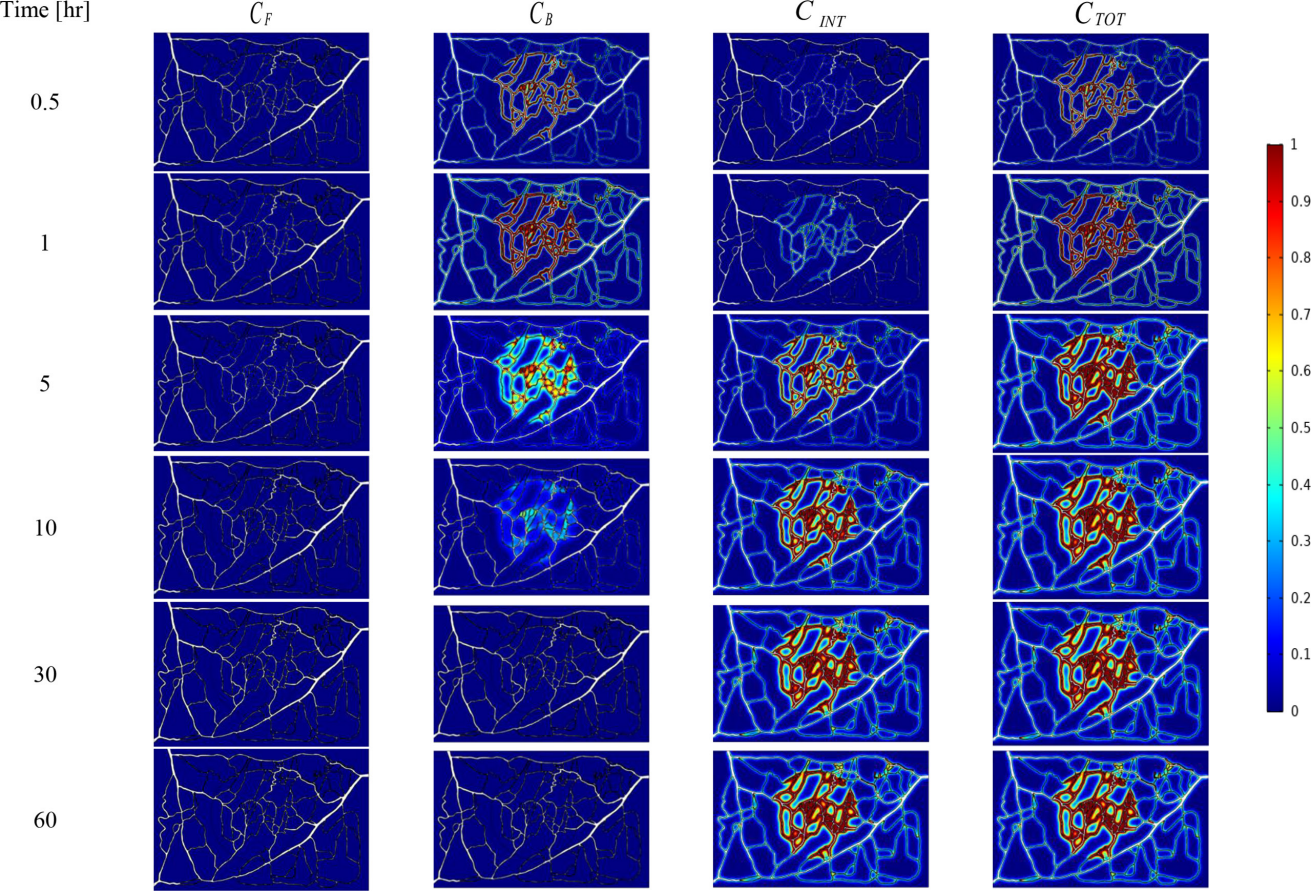
Spatiotemporal distributions of concentrations of chemotherapy drug in tumor and its surrounding normal tissue with increasing time. These non-dimensional concentrations ${\tilde{C}}_{INT}$ were calculated at a given time by dividing that concentration at any point of geometry by the maximum concentration in the whole domain.

**Figure 6:**
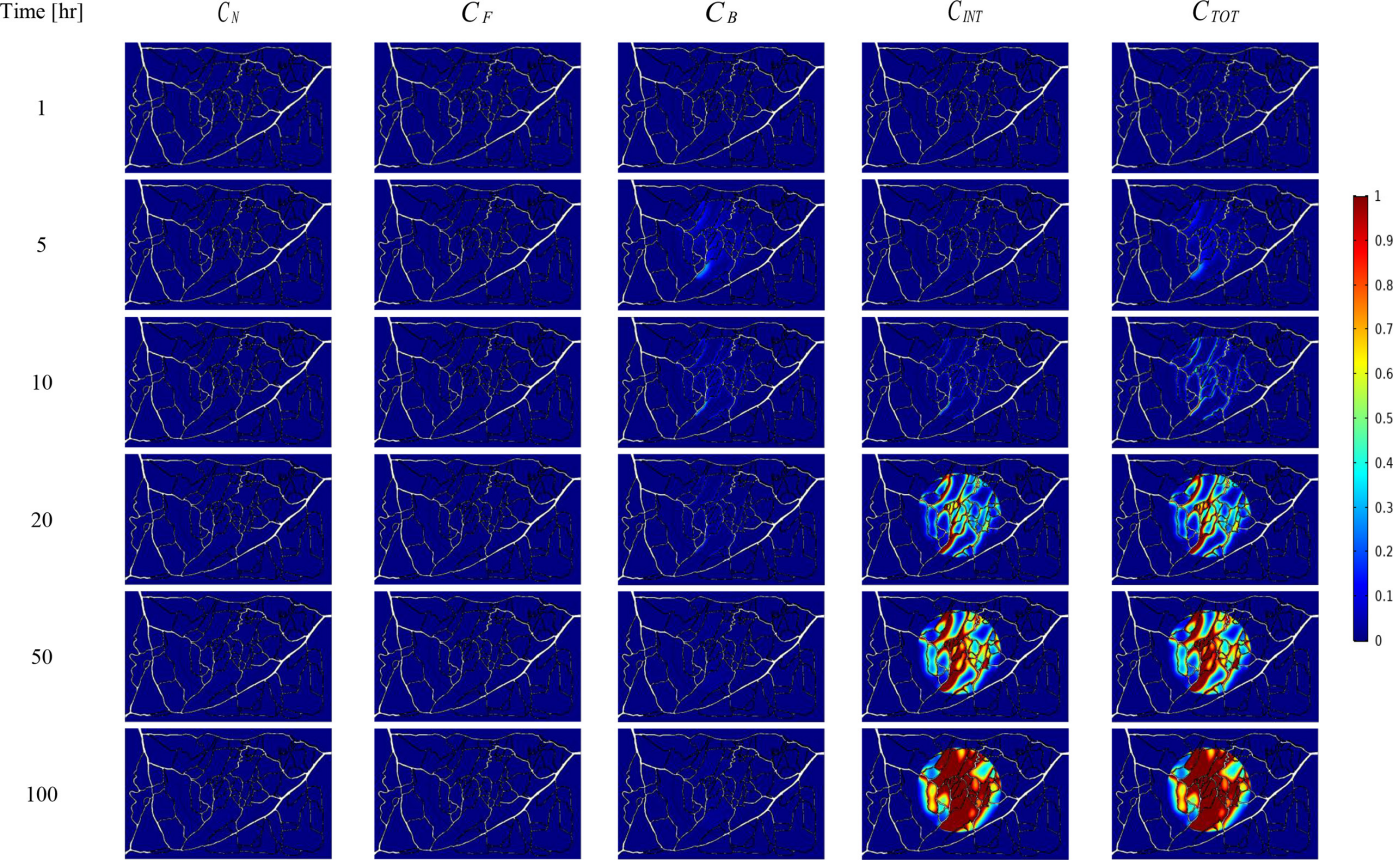
Spatiotemporal distributions of concentrations of drug-loaded NPs in tumor and its surrounding normal tissue with increasing time.

**Figure 7:**
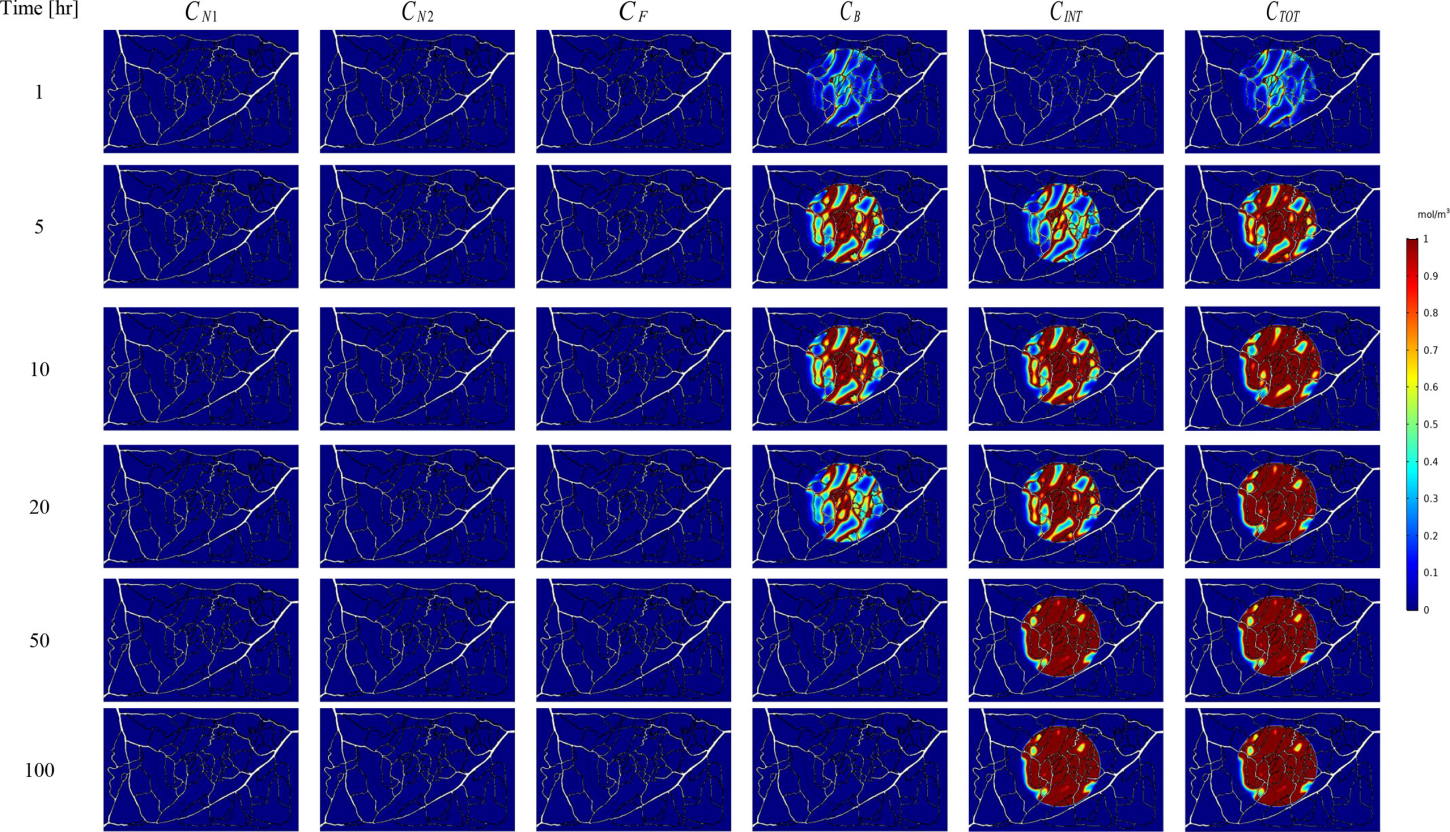
Spatiotemporal distributions of concentrations of three-stage NPs in tumor and its surrounding normal tissue with increasing time.

The spatial-temporal distribution of the non-dimensional concentrations of drug that is taken up by TCs in the tumor and surrounding normal tissue for three investigated DDSs ─single-stage (conventional chemotherapy), two-stage, and three-stage ─ are shown in **Figures 5**–**7**. These non-dimensional concentrations $\left(\tilde{C_{i}}\right)$ were obtained at a given time by dividing that concentration at any point by the maximum concentration in the entire field. Total concentration is defined as the sum of various drug concentrations. In single-state DDS, free drug concentration reduces over time and the drug gradually turns into a bound drug. Then, the bound drug slowly enters the intracellular space and is consumed there. It is also obvious that drug concentration in the tumor is greater than that in the normal tissue. The reason for this is the greater extravasation rate from microvasculature in the tumor area and additionally the highly-dense MVD in this region. The highest value for total concentration takes place in the tumor zone and this value is several times higher than the concentration in the normal tissue. In two-stage DDS, the loads of NPs are released in the interstitium in a free drug form and the remaining process is similar to single-state system. In three-stage DDS, the primary NPs release the second NPs, and the remaining process is similar to previous two-stage DDS.${\tilde{C}}_{INT}$ represents the internalization of drugs to the cellular space, determining the succeed of drug delivery in killing cancer cells (*i.e.*, leading to higher FKCs). Another important factor for efficient drug delivery is uniform distribution of drug in tumor, expressing the penetration depth of drugs extravasated from microvascular network. Comparison of the distribution of different drug concentrations in three investigated systems demonstrates that three-stage DDS provides much more uniform drug distribution than the other two ones. After that, two-stage system has more efficient drug distribution compared to single-stage DDS. From **Figures 5**–**7**, it is also clear that in conventional chemotherapy, there exist a small concentration of drug in healthy tissue, leading to side effects; whereas, in both NSDDSs, the side effects are negligible.

### Analysis of NP Drug Delivery System

Parameters of the TME that inhibit the delivery of NPs into the tumor include the size-dependency of the transport both across the tumor microvessel wall and then *via* the interstitial space of tumor. Transport across the tumor vessel wall is determined by the relative size of the particle compared to the VWP size. On the other hand, not only the size but also other parameters of drug, such as the drug release kinetics might play a crucial role in the outcome of the therapy. Therefore, one of the main goals of the present study is to determine under what circumstances two-stage and three-stage DDSs can be beneficial, relative to one another or to conventional chemotherapy. In the following, the results are presented for two- and three-stage DDSs. Then, a parameter study is carried out to examine the effect of three important parameters ─size of NPs, binding affinity, release rate of drug─ on the real geometry of tumor.

#### Two-Stage Drug Delivery System

**Figure 8:**
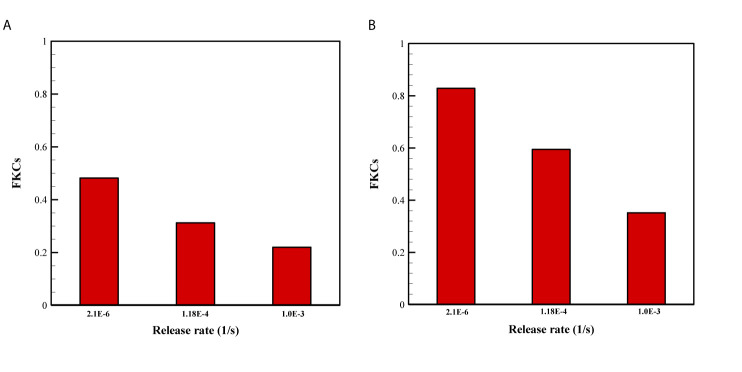
Comparison of treatment efficacy of two NP sizes for different release rates. K_ON_ was set to 15[m^3^/[(mol.s)] in all cases. **(A)** 100 nm, **(B)** 20 nm.

The effect of release rate of drug for two sizes of NPs are demonstrated in **Figure 8**. Overall, FKCs for NPs with the size of 20nm is higher than that of 100nm, in all investigate release rates of drug. The main reason is the greater blood half-life of smaller NPs compared to larger ones. Therefore, a 20nm NP is expected to circulate in the blood for a longer time in comparison with a 100nm particle, further improving efficacy of delivery systems. Moreover, it is demonstrated that slower and continuous release of the chemotherapeutic agents from NPs have better treatment results compared to faster release rate.

NP delivery systems may have other functions in addition to acting as carriers of drug. One of these functions is to control the release rate of therapeutic agents from the NPs (2). Release rate from drug-loaded nanocarriers directly determines the toxicity and anticancer activity of a DDS. Depending on various factors such as the NP formulation, fabrication method, surrounding environment, *etc.*, release rate can vary across a wide range. For instance, stealth liposomes can provide sustainable release over weeks, while TSLs are designed to release their payloads in a short time. Hence, to cover all these wide ranges, the release rate is changed from 2.1×10^-6^ to 1×10^-3^ [1/s] in this study. A very rapid drug release before the NPs have penetrated deep into the tumor may result in non-uniform drug distribution, while a very slow release may cause most NPs to be cleared out before reaching the tumor as well as it may cause multi-drug resistance (29). Moreover, release rate of drug should be regulated based on maximum efficacy in TC kill and minimum side effects. Sustained release with the goal of drug delivery over a long period of time is important for drugs that are rapidly metabolized and excreted. Sustained release can stabilize plasma concentrations of the drug at a constant level, thus reduces the need for higher doses of the drug, which results in reducing side effects.

#### Three-Stage NP Drug Delivery System

In addition to enhancing the tumoral penetration depth, multi-stage NP delivery systems can provide further tunability in the spatial delivery control to solid tumors (8, 28). Due to the physiological obstacles that a chemotherapeutic agent must encounter, a multi-stage DDS can improve treatment efficacy by altering its physical properties (including shape, size, flexibility, charge, and/or surface coating) to suit the transport across each obstacle (8). As interest in increasingly complicated DDSs grows, we face a corresponding challenge to set the model parameters. In this study, we had presented two common scenarios for multi-stage DDS: (*i*) a 100-nm particle, which released secondary 10-nm particles; and (*ii*) a 20-nm particle, which released secondary 5-nm particles.

**Figure 9:**
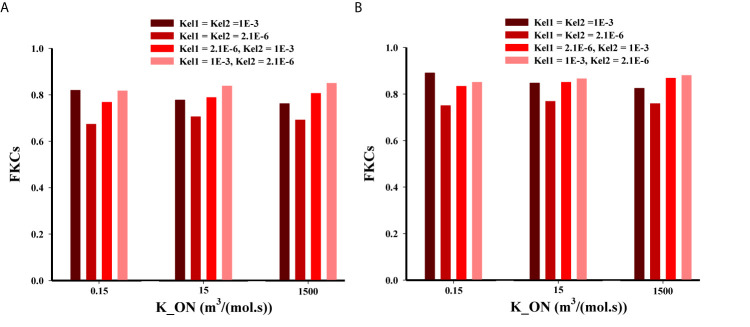
Comparison of treatment efficacy of two multi-stage delivery scenarios for different values of K_ON_, K_el1_, and K_el2_. **(A)** NP1=100nm and NP2=10nm, **(B)** NP1=20nm and NP2=5nm.

Based on **Figure 9** for the three-stage DDSs, we assumed multiple different possible states for release rate constants (*i.e.*, K_el1_ and K_el2_) of the secondary particle and the drug. The results enable a set of observations as to how release rate constants affect the efficacy of drugs. The four most predominant are: (*i*) overall, the second scenario (NP1 = 20nm and NP2 = 5nm) has higher treatment efficacy, almost in all investigated states, compared to the first scenario (NP1 = 100nm and NP2 = 10nm); (*ii*) in low binding rates, the high release rates have better performance; (*iii*) in moderate and high binding rates, the NP release must have high release rates and the drug release must have the lower release rates; (*iv*) the least treatment efficacy occurs when both release rates are slow.

A high binding affinity of drug agents to receptors of cancer-cell leads to a higher drug concentration in the intracellular space of tumor, and it simultaneously decreases the drug concentration in tumor tissue. In other words, with a higher binding affinity, more drugs are taken up by cancer cells, reducing the drug level in the interstitium. Binding affinity plays different roles in the delivery of smaller or larger particles because in smaller particles the diffusion mechanism is more dominant than convection, while in larger particles the convection *via* vessel-walls is more dominant in the transport across blood vessels. It should be mentioned that a very high binding affinity of the NPs leads to aggregation nearby the vessels that NPs are extravasated from. On the other hand, for transport from microvessels to tissue, NP efficacy depends on the kinetics of drug release from particles (28). Eventually, the released drug may also rapidly bind to the cells, causing heterogeneous and incomplete distribution within tumor (62). After simultaneous investigation of the impacts of NP size, binding affinity, and the release rate of drugs, it is predicted that NP penetration from tumor microvessels would be affected (29). Indeed, there is a competition between diffusion in the ECM (which depends on NP size) and binding affinity and/or the release rate of drugs. However, the one of the main conclusions is that all these three parameters should be considered together and studying their impact alone does not provide the right policy on optimal design of NSDDSs.

### Treatment Evaluation

**Figure 10:**
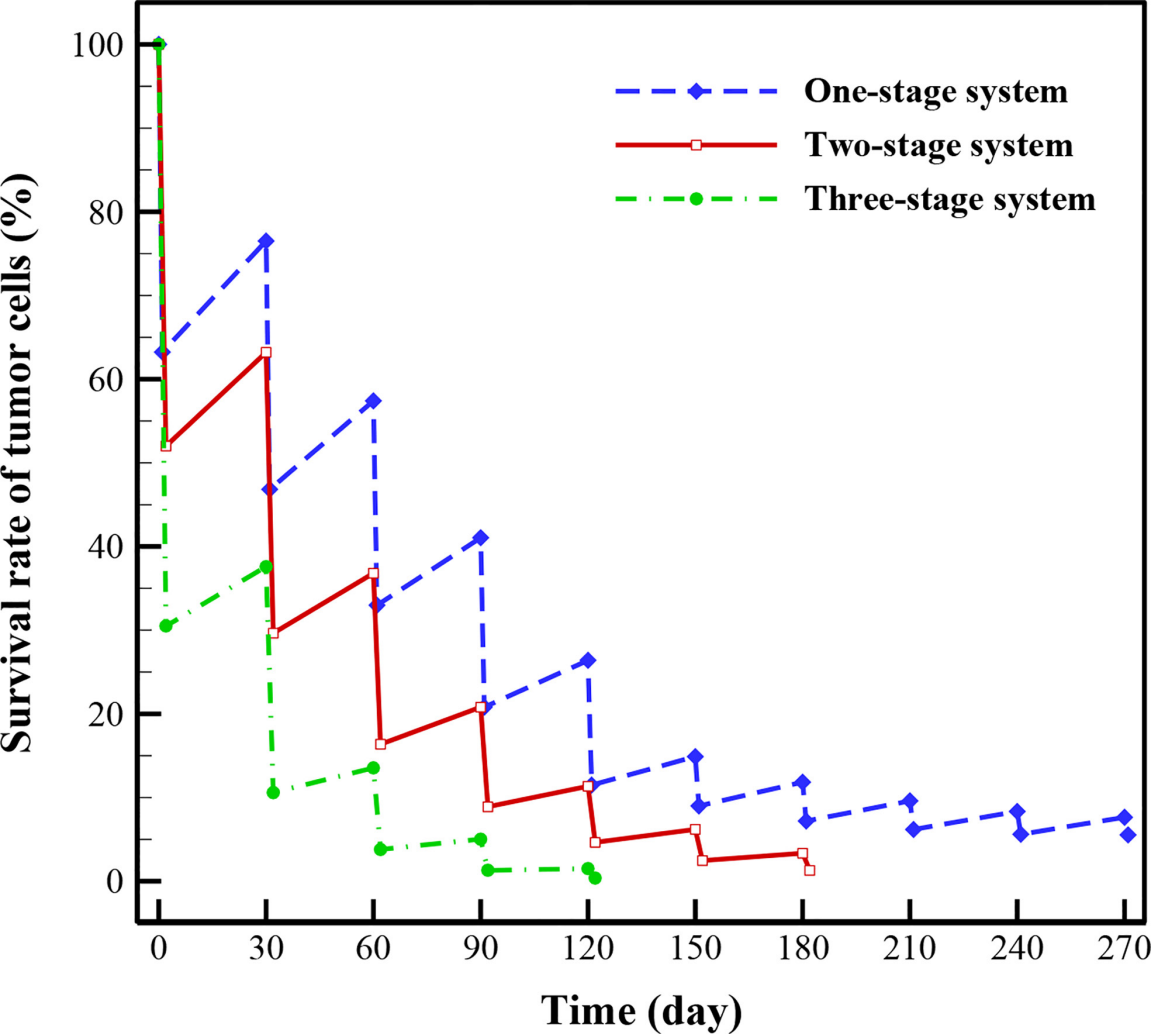
Survival rate of TCs for one (conventional), two and three-stage drug delivery systems.

In this section, the treatment outcomes of three investigated drug delivery systems are calculated by considering 1-month drug-free breaks between treatments. As demonstrated in **Figure 10**, three-stage system has better treatment outcome than two-stage and one-stage (i.e., conventional chemotherapy) systems, implying superiority based on higher rate of killing TCs and shorter treatment time, simultaneously. For the conventional chemotherapy, the respective efficacies of the first, second, and third stages of treatment in reducing the TCs are about 36.78%, 29.67%, and 24.41%. This rate is 20.24% for the fourth stage, 14.9% for the fifth stage, 5.9% for the sixth stage, 4.65% for the seventh stage, 3.42% for the sixth stage, 2.73% for the ninth stage, and 2.11% for the tenth stage. Tumor regrowth between the first and second stages of treatment, between the second and third stages, and up to the end of the process in the tenth stage are calculated to be 13.28%, 10.57%, 8.06%, 5.59%, 3.4%, 2.85%, 2.4%, 2.17%, and 2.03%, respectively. At the last stage of treatment, it is obvious that the regrowth of TCs is about 2.03%, while the treatment efficacy is about 2.11%, implying that for this size of tumor using conventional chemotherapy alone is not the best choice and need more cycles of treatments, so an adjuvant therapy would be suggested at this size of tumor. In general, after seven treatments, 5.54% of the initial tumor cells remain for conventional chemotherapy.

Results indicate that after 5 treatments with three-stage system, 99.6% of TCs are killed, while two-stage and one-stage system respectively kill 95.6% and 88.5% of TCs in the same period. It should be mentioned that the rate of killing TCs for the two-stage system reaches 98.9% after 7 cycles of treatment. This rate is also 94.46% for one-stage system after 10 treatment cycles. As a consequence, the treatment efficacies for both the NSDDSs have shown significant improvements compared to conventional chemotherapy. Results of the case study show that the survival rates of TCs after several treatment cycles offer significant prognostic insight about the survival rate and the cell regrowth percentage. Being able to evaluate the efficacy of a treatment scenario using multi-scale computational modeling is very helpful in clinical situations.

## Conclusion

In the present work, a mathematical model of drug transport is proposed to predict delivery of chemotherapeutic drugs, either in their free form or encapsulated in NPs. Parameters of the model describing physiological and biological characteristics of tissue and drug are extracted from experimental data in the literature. The model has been applied to realistic tumor reconstructed from actual image of vascularized tumor to: (*i*) understand the transport steps of non-encapsulated and encapsulated drugs, and (*ii*) elucidate the impact of drug properties on drug delivery and treatment. The main findings are as follows:

- Spatiotemporal drug distribution illustrates that the complexity of the capillary network is the main factor for non-uniform drug distribution in the tumor.
- The FKCs of tumor for two-stage DDS with smaller size of NPs (20nm) is higher than that of larger ones (100nm), in all investigate release rates. Slower and continuous release of the chemotherapeutic agents from NPs have better treatment outcomes in comparison with faster release rate.
- For three-stage DDSs, in intermediate and higher binding affinities, it is desirable for the secondary particle to be released with faster rate, and the drug with slower rate. In lower binding affinities, the high release rates have better performance
- Three-stage system has better treatment results relative to two-stage and one-stage systems, reaching 99.6% effectiveness in killing TCs after 5 treatments; while two-stage and one-stage system respectively kill 95.6% and 88.5% of TCs in the same period.

Overall, a mathematical framework has been developed for drug delivery in both free and encapsulated forms to provide qualitative and mechanistic understanding of transport of drug in solid tumors. The effect of treatment outcomes considering tumor recurrence between the two presented models is highly complementary to PK/PD models as it considers both the spatial and temporal scales at the same time, while PK/PD models merely consider the temporal scales. Moreover, different equation parameters in the present study have physiological and bio-chemical implications (*e.g.*, intravascular pressure, microvascular density, microvascular diameter, permeability, diffusion coefficient to name a few), whereas PK/PD methods might involve a parameter to express several parameters.

It should be mentioned that anti-angiogenic agent (here, TSP2) has effects on real tumor geometry and changes of permeability, but we have no discussion about TSP2 in the present study. On the other hand, if we have three images for three different states (negative control, treatment with TSP2 and without treatment), the mathematical modeling can include the new geometry of microvascular networks. This is an interesting suggestion for future studies. Additionally, this method can be applied on each images of tumor because the geometry of tumor can be obtained after image-processing; however, in this study, we have chosen an actual image from the literature due to the lack of experimental set-up in our group.

Protection systems for NPs such as PEGylation as well as adjustable parameters of NP design, all try to enhance drug half-life in the circulation system, which can increase drug delivery into the tumor and reduce side effects to healthy tissue. Furthermore, NPs can be manipulated to release their loaded drugs if exposed to a specific external (*e.g.*, ultrasound or magnetic field) or internal (e.g., pH or enzyme) stimulus. Multi stimuli-responsive DDSs ─combination of internal and external stimuli─ have not only succeeded in targeted drug delivery but also in the multi-modal cancer diagnosis and treatment. In addition to enhancing efficacy of encapsulation, these systems may enhance the drugs half-life. Our presented approach has the potential of modeling these targeting systems. For example, by simultaneously solving bio-heat and wave equations with the presented equations, the drug delivery through thermo-sensitive NPs can be modeled.

## Data Availability Statement

The original contributions presented in the study are included in the article. Further inquiries can be directed to the corresponding author.

## Author Contributions

Data curation, FM. Investigation, FM, MS, and MM. Methodology, FM. Project administration, FM and MS. Resources, FM. Software: FM and MM. Supervision, MS and AR. Validation: FM. Visualization, FM and MM. Writing – original draft, FM. Writing – review and editing, MS, MM, and AR. Funding acquisition, AR. All authors contributed to the article and approved the submitted version.

## Funding

The authors wish to acknowledge funding from the Natural Sciences and Engineering Research Council of Canada (NSERC) Discovery Grant RGPIN-2019-06467, as well as the BC Cancer Foundation.

## Conflict of Interest

The authors declare that the research was conducted in the absence of any commercial or financial relationships that could be construed as a potential conflict of interest.
